# A joint industry‐sponsored data monitoring committee model for observational, retrospective drug safety studies in the real‐world setting

**DOI:** 10.1002/pds.5172

**Published:** 2020-11-24

**Authors:** Atheline Major‐Pedersen, Mary Kate McCullen, Mary Elizabeth Sabol, Omolara Adetunji, Joseph Massaro, Alfred I. Neugut, Julie Ann Sosa, Anthony N. Hollenberg

**Affiliations:** ^1^ Global Safety, Novo Nordisk A/S Copenhagen Denmark; ^2^ US Patient Safety Surveillance, AstraZeneca Wilmington Delaware USA; ^3^ Safety Evaluation & Risk Management, GlaxoSmithKline Philadelphia Pennsylvania USA; ^4^ Global Patient Safety, Eli Lilly and Company Windlesham UK; ^5^ Department of Biostatistics Boston University School of Public Health Boston Massachusetts USA; ^6^ Departments of Medicine and Epidemiology Columbia University Medical Center New York New York USA; ^7^ Department of Surgery University of California San Francisco (UCSF) San Francisco California USA; ^8^ Joan and Sanford I. Weill Department of Medicine Weill Cornell Medicine, New York‐Presbyterian Hospital‐Weill Cornell Medical Center New York New York USA

**Keywords:** data monitoring committee, joint sponsorship, observational, real‐world data, retrospective

## Abstract

**Purpose:**

To share better practice in establishing data monitoring committees (DMCs) for observational, retrospective safety studies with joint‐industry sponsorship.

**Methods:**

A DMC model was created to monitor data from an observational, retrospective, post‐authorization safety study investigating risk of medullary thyroid cancer in patients treated with long‐acting glucagon‐like peptide‐1 receptor agonists (LA GLP‐1RAs) (NCT01511393). Sponsors reviewed regulatory guidelines, best practice and sponsors' standard operation procedures on DMCs. Discussions were held within the four‐member consortium, assessing applicability to observational, retrospective, real‐world studies. A DMC charter was drafted based on a sponsor‐proposed, adapted DMC model. Thereafter, a kick‐off meeting between sponsors and DMC members was held to receive DMC input and finalize the charter.

**Results:**

Due to this study's observational, retrospective nature, assuring participant safety – central for traditional explanatory clinical trial models – was not applicable to our DMC model. The overall strategy and key indication for our real‐world model included preserving study integrity and credibility. Therefore, DMC member independence and their contribution of expert knowledge were essential. To ensure between‐sponsor data confidentiality, all study committees/corporations and sponsors, besides the DMC, received blinded data only (adapted to refer to data blinding that revealed the specific marketed LA GLP‐1RA/sponsor). Communication and blinding/unblinding of these data were facilitated by the contract research organization, which also provided crucial operational oversight.

**Conclusions:**

To our knowledge, we have established the first DMC model for joint industry‐sponsored, observational, retrospective safety studies. This model could serve as a precedent for others performing similar post‐marketing, joint industry‐sponsored pharmacovigilance activities.

**Key points**

- Observational, retrospective studies are increasingly performed to assess real‐world drug safety. They are important tools for detecting rare events (e.g. medullary thyroid cancer [MTC]) that are difficult to measure in drug development programs.
- Stakeholders (regulatory health authorities, payers, physicians) request real‐world data to support clinical trial data (internal validity) with external validity.
- Data monitoring committees (DMCs) may play a central role in validation and ongoing interpretation of the increasingly extensive data emerging from real‐world studies.
- Our DMC model was shown to be effective for an observational, retrospective, joint industry‐sponsored study.
- This model may be of interest to others involved in this evolving, real‐world pharmacovigilance discipline, particularly in challenging/rare diseases such as MTC.

## 1 INTRODUCTION

The importance of real‐world evidence to support clinical trial data is increasingly recognized and requested by stakeholders, for example, health regulatory agencies, health policy decision‐makers, patient groups, public funders and academia. Clinical trial limitations may include short duration, small populations and selective inclusion criteria. Moreover, results from highly selected and controlled environments provide high internal validity but are not necessarily generalizable to real‐world settings (i.e. failure to provide external validity).^1^, ^2^, ^3^, ^4^, ^5^ Consequently, the potential existence of unforeseen safety signals/safety concerns (unknowns) may persist, despite evaluation in well‐performed clinical trials and following marketing authorization.

Industry sponsors with marketing authorizations for same‐class drugs are encouraged, by regulatory authorities, to conduct joint collaborative real‐world studies.^6^ Hence, inconvenience to patients, physicians and registries is minimized while data exposure is maximized. Increasing recognition of the value of drug safety studied in such settings has resulted in comprehensive databases that may warrant enhanced monitoring.

Data monitoring committees (DMCs) were first described in 1967.^7^ Despite increased and evolved use over the last 50 years, DMCs remain within the clinical trial realm. We believe joint multi‐disciplinary and multi‐institutional efforts are needed to establish robust infrastructures supporting real‐world studies, including DMC empowerment beyond clinical trials, lending study integrity and credibility to real‐world studies. The DMC model we share monitors data from an observational, retrospective, real‐world study. This model accommodated additional challenges resulting from one DMC serving four industry sponsors within one study.

## 2 METHODS

### 2.1 Study setting

This DMC model aimed to monitor safety data from an observational, retrospective, United States (US)‐based, jointly sponsored, post‐authorization safety study (PASS) investigating the risk of medullary thyroid cancer (MTC) in adults treated with long‐acting glucagon‐like peptide‐1 receptor agonists (LA GLP‐1RAs) (NCT01511393). This study was required by the FDA due to findings of dose‐related and treatment duration‐dependent increases in the incidence of thyroid C‐cell tumors (adenomas and carcinomas) in rodents at clinically relevant doses of GLP‐1 RAs. The clinical relevance of rodent thyroid findings observed with GLP‐1 RAs remains unknown. Due to MTC rarity (incidence: 0.2/100,000)^8^ and patient, physician and registry inconvenience, the Food and Drug Administration (FDA) required all sponsors with marketed LA GLP‐1 RA products to collaborate, resulting in a consortium currently comprising four industry sponsors (AstraZeneca; Eli Lilly and Company; GlaxoSmithKline; Novo Nordisk A/S, Denmark). For each compound, MTC registry continuation was anticipated for 15 years post FDA‐approval (or a sponsor‐FDA agreed duration). First FDA approval for a sponsor's LA GLP‐1 RA was in 2010.

### 2.2 Process and tools

DMC regulatory guidelines, DMC best practice and each sponsor's DMC standard operation practices (SOPs), available at the time of DMC charter drafting, were reviewed regarding: (i) precedence for DMC establishment for observational, real‐world studies based on secondary data use; (ii) precedence for joint industry‐sponsored studies; (iii) indication for DMC; (iv) DMC member independence and composition; (v) safety data for DMC review (including statistical analysis plan [SAP]); (vi) conduct and structure of DMC data review meetings; (vii) DMC recommendations to the sponsor; (viii) DMC communication flow with sponsors and Steering Committee; (ix) kick‐off meeting–charter finalization. For the MTC registry study itself, the 28 participating state registries were required to obtain institutional review board, relevant department of health or ethics committee approval of the MTC registry document/project manual prior to inclusion of cases in the registry.

Sponsors held discussions internally and collectively (within the sponsor consortium) regarding: (i) applicability of DMC guidelines/best practice recommendations/SOPs to a DMC model for observational, retrospective real‐world studies; (ii) need for adaptations; (iii) consensus on suggested DMC‐related process adaptations. The sponsor consortium also collaborated with members of the American Thyroid Association (ATA), a professional organization comprising thyroid cancer specialists, whose members participated in consortium meetings. Thereafter, a DMC charter was drafted based on the sponsor‐proposed adapted DMC model. The first charter draft was reviewed by all sponsors participating in the study at the time. Sponsors joining the study later were included in successive charter reviews.

Sponsors held a kick‐off meeting for all DMC members and sponsor representatives to discuss the DMC charter and SAP drafts and implement comments. Post meeting, the DMC charter was finalized; DMC member comments were implemented after internal and between sponsor agreement and a subsequent signature round including the DMC chair, contract research organization (CRO) and all sponsors.

## 3 RESULTS

The following results are set out according to (i) findings following review of the DMC guidelines/best practice, as detailed in the Methods (Section 2.2); and (ii) applicability of these “recommendations” to our DMC model (also summarized in Appendix S1).

### 3.1 Precedence according to DMC guidelines and best practice

Health regulatory and government funding agencies created DMC guidelines (henceforth, referred to as “guidelines”)^9^, ^10^, ^11^, ^12^, ^13^ based on the Greenberg DMC model.^7^ Like the Greenberg model, guidelines focused on DMCs for explanatory clinical trials. Lilienfeld et al. recognized necessary DMC role evolution from pre‐clinical to post‐marketing trial settings, including observational studies.^14^ However, no solid experience with their proposed DMC model was shared. Except for a DMC acting in a prospective, open‐label, observational study,^15^ authors have described and discussed consensus and controversies of the DMC set‐up for traditional explanatory clinical trial settings.^16^, ^17^, ^18^, ^19^, ^20^, ^21^, ^22^, ^23^, ^24^ Hereafter, these are referred to as “best practice.” Only explanatory clinical trials were in scope for each sponsor's DMC SOPs. Although there is precedence for a single DMC reviewing multiple trials,^22^, ^25^ these involved only one sponsor. Hence, no best practice for a joint industry‐sponsored DMC model was identified.

#### 3.1.1 Applicability

No guidance/sponsor SOPs for DMCs serving observational, retrospective studies exist, and no solid experience regarding such DMCs has been shared; thus, there was no best practice precedence.

### 3.2 Indication for DMC according to DMC guidelines and best practice

Safeguarding the interests and safety of trial participants was a central indication for creating a DMC, particularly relevant for trials where the studied population was fragile, vulnerable or at elevated risk of serious outcomes/death, the studied disease was life‐threatening, or the studied treatment was considered invasive/toxic. Other indications for DMCs include preserving trial integrity (scientific validity) and credibility, particularly for large, multicenter, long‐duration trials, where interim analyses might ethically require trial termination before planned completion (e.g. due to futility), or trials with complex designs requiring potential modifications, depending on unblinded interim data.

#### 3.2.1 Applicability

Assuring trial participants' safety was not applicable to our DMC model because the “event of interest” had already occurred when drug exposure data (prior to the studied outcome) were captured in the retrospective studies (i.e. a previous diagnosis of MTC was an inclusion criterion).

Preserving study integrity and credibility was deemed the main indication for our model. The increasing complexity of real‐world data from observational, retrospective studies requires expertise for appropriate analysis and interpretation. Furthermore, changes in understanding of the disease, the affected population and standard real‐world treatment may happen over the 15+ years of our study, warranting independent DMC oversight.

The pre‐determined possibility of changing the study design based on interim analyses was identified as an important indication for our DMC model. Regulatory‐imposed observational, retrospective PASSs are usually required to perform and submit annual interim analyses, thus creating the possibility of study design modification.

### 3.3 DMC member independence and composition according to DMC guidelines and best practice

#### 3.3.1 Independence

All DMC members should be academically and financially independent of the trial's outcome, sponsor and Steering Committee to review the emerging data in an unbiased fashion. Some have recommended that DMC members should be convened by a third party, such as an independent professional body^23^ (e.g. ATA).

DMC independence was considered especially important to ensure patient safety, as well as necessary to avoid “real or perceived conflict of interest/bias,” thus ensuring the credibility of DMC decisions and study integrity. The statistician evaluating the interim analyses of unblinded data should be independent of the sponsor and a non‐voting member of the DMC, additional to the DMC voting statistician.

#### 3.3.2 Composition

The DMC should include medically qualified clinical trial experts within the areas relevant to the study's population and outcomes. Additional members should include statisticians, ethicists and patient advocates from patient populations using the product. Previous DMC experience is also desirable.^20^, ^22^ Lilienfeld et al. also recommended that real‐world studies encompass expertise within pharmacoepidemiology, health education and program evaluation.^14^ The number of members should be limited to 3–7, in accordance with a median number of 4 (range 3–20).^17^

#### 3.3.3 Applicability

DMC members in our model do not protect study participant safety. Nevertheless, DMC independence was deemed highly relevant for providing study credibility and study integrity. Identifying sponsor‐independent candidates was challenging because all four industry sponsors had a long history of collaboration with many key experts within the same therapeutic area (endocrinology). Furthermore, each sponsor was familiar with their own SOPs when working with external experts. Having a CRO acting as facilitator between sponsors and between sponsors and potential DMC candidates reduced process ownership of any one sponsor and enhanced inter‐sponsor dialog regarding necessary compromises. In hindsight, sponsors could have delegated identification of potential DMC candidates to the CRO or independent professional body (e.g. ATA), thereby further enhancing DMC sponsor independence and simultaneously simplifying inter‐sponsor communication.

**FIGURE 1:**
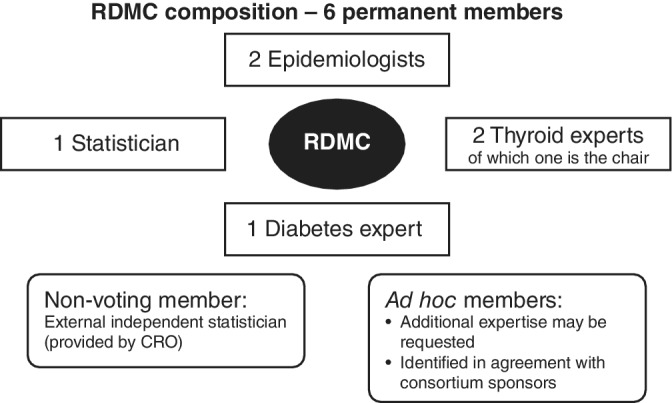
DMC model composition. Legend: CRO, contract research organization; DMC, Data Monitoring Committee; RDMC, Registry DMC

The DMC model was adapted to include two epidemiologists and a statistician with expertise in observational study designs (Figure 1). Including patient advocates and data‐programming experts could also have been relevant for this DMC model reviewing real‐world data.

All DMC members received travel reimbursement and honoraria from the sponsors via the CRO.

### 3.4 DMC roles and responsibilities according to DMC guidelines and best practice

Trial participant safety was considered the primordial DMC role and responsibility. Other proposed DMC goals were related to study conduct and progress, including patient recruitment, protocol compliance and data quality.^16^ Hicks et al. recommended assigning trial oversight to others involved in the study so that the DMC could “focus on important outcomes that might justify early study termination or modification.”^22^

#### 3.4.1 Applicability

As discussed, trial subject safety was not applicable to our DMC model. Aligned with Lillenfeld et al,^14^ we found that responsibility of a DMC (monitoring real‐world data) is to patients, physicians, payers, health policy regulators and others who are impacted by having the pharmaceutical approved. Our model focused on issues that may justify modifying study design or earlier FDA reporting (see Section 3.7.1, DMC recommendations). Therefore, study conduct (which includes data quality assessment) and progress oversight were transferred to the study's Steering Committee and sponsor consortium, supported by the study CRO. The sponsor consortium, together with ATA experts, agreed that the DMC would not perform adjudication of MTC cases.

### 3.5 DMC review of safety data according to DMC guidelines and best practice

DMCs should have access to all available clinical trial data, including unblinded data in treatment‐blinded studies. A SAP should be established before the DMC initiates data analysis. Best practice documents discussed the advantages and disadvantages of: (i) DMC access to blinded data from other clinical trials' DMCs investigating the same drug/drug class; (ii) a single DMC for all trials within the same drug/drug class, within the same company; (iii) regulatory authority access to a DMC's analysis of blinded interim data before study closure.

#### 3.5.1 Applicability

Blinding of treatment arms was not applicable to our DMC model; there was no treatment randomization and neither physicians nor patients were blinded to treatment prior to MTC diagnosis. We adopted and adapted the terms “blinded” and “unblinded” data. “Blinded” in this study referred to blinding of data that revealed the specific marketed LA GLP‐1RA/sponsor. To retain sponsor confidentiality, sponsors and all other study committees/corporations (except for certain CRO employees) received only blinded data comprising safety data for LA GLP‐1RAs as a class, and not the individual drug. Each sponsor received unblinded data related to its own drug from the CRO, enabling ongoing and timely pharmacovigilance activities.

The unblinded data pack provided to the DMC included specific LA GLP‐1RA generic and/or commercial names and the sponsors' names. Data packages presented in DMC‐requested formats were prepared and delivered via a secure portal by the external independent statistician (provided by the CRO) approximately 3–4 weeks prior to each DMC meeting on an annual basis. Interim analyses containing blinded data and study progress were submitted annually to the FDA.

Whether to share the DMC's blinded analyses with other DMCs examining the same safety concern has not been applicable to our DMC model to date. Our model reviewed all accumulating safety information on MTC risk related to LA GLP‐1RA exposure across all sponsors with a marketed drug within the drug class. Additionally, the DMC received information annually from each study sponsor for all MTC cases (spontaneous and solicited reports) from other sources globally.

### 3.6 DMC data review meetings according to DMC guidelines and best practice

Open and closed DMC sessions were described, based on the model initially introduced by the National Institutes of Allergy and Infectious Diseases (NIAID) and the National Institutes of Health (NIH) NIAD Acquired Immunodeficiency Syndrome (AIDS) Clinical Trials Group, which also included an executive committee session.^11^, ^12^, ^25^ No consensus was reached, across the guidelines and best practice, as to whether only DMC members should be allowed to attend the closed sessions. The meeting venue should be in a neutral location.

#### 3.6.1 Applicability

**FIGURE 2:**
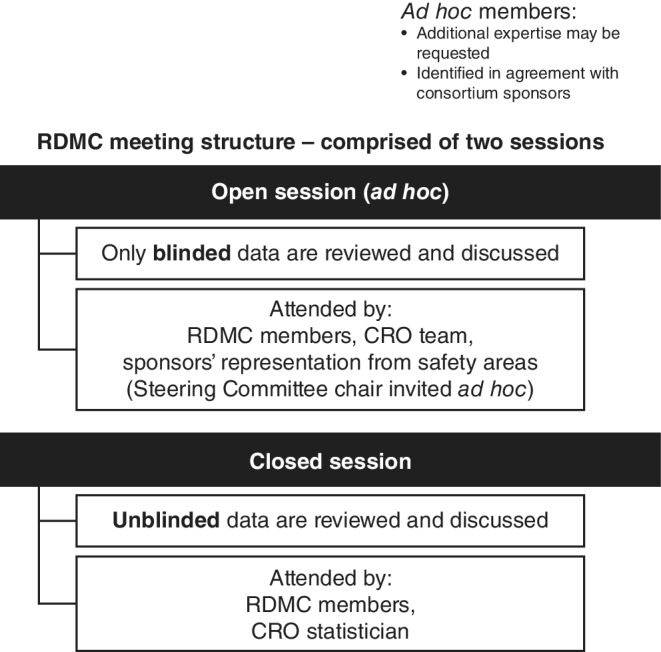
RDMC meeting structure, comprising open and closed sessions. Legend: CRO, contract research organization; RDMC, Registry DMC

The open and closed sessions were adapted to our DMC model to align with the model‐defined concept of “blinded” data (Figure 2).

DMC members and sponsors attended open sessions, since only blinded data were reviewed. To maintain DMC independence and reduce sponsor interactions with DMC members, sponsors and the DMC kept open sessions as an ad‐hoc possibility. Sponsor representatives could attend the open session via teleconference, facilitated by the CRO, while the DMC members met face‐to‐face. To date, five DMC meetings have taken place (one DMC meeting annually) in which DMC members have met, while sponsor representatives for the four sponsors participated via teleconference in the open session which preceded each of the five closed sessions. DMC members are reminded at the beginning of each open session to refrain from commenting on sponsor‐specific data (blinded data) during sessions. Steering Committee members also could be invited to the DMC open session, if relevant.

Closed sessions were attended exclusively by DMC members (including the non‐voting, external, independent CRO statistician) where unblinded data (specific LA GLP‐1RA exposure drug and company are revealed) were reviewed and discussed. Moreover, during closed sessions, the DMC could request external ad‐hoc consultation from the CRO statistician (without sponsor involvement), unscheduled teleconferences with one or more of the sponsors via the CRO, ad‐hoc meetings or additional data.

Considering its complex structure, it was not deemed necessary to incorporate an executive committee session into our DMC model. The meeting venue had to fulfill each of the four sponsors' SOPs, applying the SOP with the strictest criteria for interacting with external experts and healthcare representatives.

### 3.7 DMC recommendations according to DMC guidelines and best practice

To ensure trial participant safety, the DMC should make formal recommendations to clinical trial sponsors after review of interim data, such as: (i) continue without modifications; (ii) stop wholly or partly; (iii) continue with modifications. Stopping a trial early could be due to futility or a positive treatment effect. Study design modification could be due to an unblinded interim data review.

#### 3.7.1 Applicability

The recommendation of stopping a study to ensure patient safety was not applicable to our DMC model. Indeed, a significant finding might warrant continuing the study until adequate knowledge is obtained, as a DMC responsibility to societies where the drugs already have been marketed.

Recommendations in our DMC model reflected the study's ongoing validity (futility) and overall credibility of results. Based on the above considerations, and to reflect the retrospective nature of the collected data emerging from the real‐world setting, the recommendations shown in Box 1 were incorporated into our model and signed by the DMC chairman after each closed session.

BOX 1 **Excerpt from the DMC recommendation template – Appendix in the finalized DMC charter**

It was decided to recommend that the

Medullary Thyroid Carcinoma Surveillance Study: A Case‐Series Registry should

- Continue unaltered
- Continue with modifications
- Report to the FDA with earlier than the next planned annual report submission

Date of signature: _____________________DD‐MM‐YYYY.

### 3.8 DMC communication flow to sponsors following data review meetings as recommended by DMC guidelines and best practice

DMC recommendations could be communicated directly to the trial sponsor or a study steering group/third party. The communication should preferably be in writing, maintaining blinded data confidentiality.

#### 3.8.1 Applicability

Due to the added complexity inherent in a DMC reviewing safety data related to multiple sponsors' study drugs, the above was not directly applicable to our DMC model.

DMC communication to sponsors could not reveal other study sponsors' confidential (blinded) data. However, if a safety concern was raised with a study drug(s)/sponsor(s), the remaining study sponsors had to be informed promptly to perform timely pharmacovigilance activities.

**FIGURE 3:**
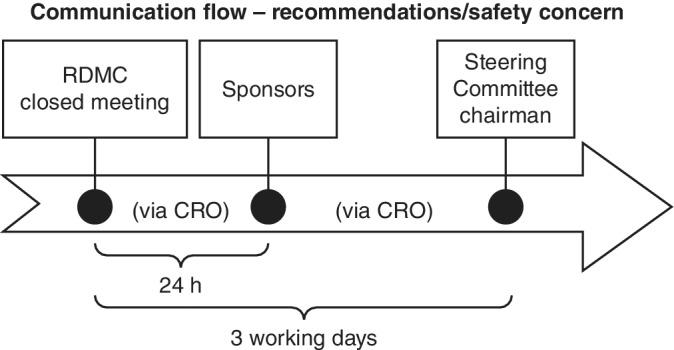
Agreed communication flow for raising DMC recommendations and safety concerns. Legend: CRO, contract research organization; DMC, Data Monitoring Committee; RDMC, Registry DMC

The sponsors, DMC members, Steering Committee and CRO repeatedly discussed different communication flow scenarios that could fulfill these factors, agreeing on the communication flow depicted in Figure 3. DMC recommendations would be communicated to sponsors via CROs within 24 h of the DMC closed session, using the DMC recommendation template. Similarly, safety concerns raised by the DMC would be communicated to all study sponsors via the CRO within 24 h of the DMC closed session. To maintain sponsor confidentiality, the agreed wording to be used by the DMC chair informing study sponsors of a safety concern was “[*Safety concern XXX*] was identified for one or more LA GLP‐1 RAs.”

Each consortium sponsor would facilitate the appropriate actions according to each sponsor's internal SOPs to support their pharmacovigilance obligations. Also, each sponsor responsible for one or more of the drugs triggering the safety concern would be directly informed by the chairman, facilitated by the CRO. As a minimum, if safety concerns/signals (and/or recommendations to continue the study with modifications, or to report to the FDA earlier than the next planned annual report submission) were raised by the DMC, a dialog between the DMC chair, sponsor representatives (according to each company's internal procedure) and the CRO would take place before further actions and decisions were made. The written communication on DMC recommendations/raised safety concerns would be conveyed to the Steering Committee chair by the CRO at the end of the third working day after the closed session. In our model, the sponsors had final responsibility for acting on the DMC's recommendations.

### 3.9 DMC kick‐off meeting – DMC charter and SAP according to DMC guidelines and best practice

The DMC set‐up should be described in a document (e.g. DMC charter) before the DMC initiates trial data monitoring. DMC charter templates have been published.^17^, ^19^ It was proposed that DMC charters be considered checklists, acknowledging that DMC models vary according to trial nature.^20^ An early DMC meeting was recommended, allowing members to meet and provide input to the DMC charter prior to its finalization.

#### 3.9.1 Applicability

We introduced a “sponsor pre‐DMC kick‐off meeting” to our model. Due to the study's joint industry sponsorship, this meeting was to establish house rules for the ensuing DMC meeting and ensure all sponsors were aligned regarding interpretation of the latest DMC charter version, SAP and study protocol.

The DMC kick‐off meeting happened prior to initiation of DMC data monitoring activities, as recommended. There was opportunity for DMC members to contribute to all charter key points. The DMC charter was finalized after the kick‐off meeting (see Appendix S2 for a template, based on our charter). Following finalization, five DMC data review meetings were conducted. The DMC's recommendation after each meeting was to continue the study unaltered.

## 4 DISCUSSION

We believe ours is the first DMC model for observational, retrospective studies, and is also applicable to joint industry‐sponsored studies. Best practice publications, available post‐charter finalization for this DMC model, remain focused on clinical trial DMCs.^26^, ^27^, ^28^, ^29^, ^30^, ^31^, ^32^ Although not of an observational or retrospective nature, Ellenberg et al. have conceptualized moving the explanatory clinical trial DMC model to the real‐world setting, and discuss challenges in applying the traditional model to pragmatic clinical trials.^33^

Several challenges were met during model set‐up, including a lack of DMC precedents for retrospective, observational studies and necessary multi‐sponsor collaboration, each with respective SOPs. Our model includes several essential factors for efficient operation, including: expert knowledge provided by DMC members; close sponsor‐consortium and ATA collaboration; and CRO operational oversight (between all sponsors, DMC and Steering Committee) and facilitation of blinded and unblinded data‐flow.

The model set‐up has the disadvantage of precluding counseling on arising study conduct issues from the DMC members, who are highly knowledgeable on the matter under investigation. This disadvantage is offset by ensuring DMC independency. DMC independence of study conduct and results and of study progress oversight enhances credibility that safety data are reviewed in an unbiased fashion. This is especially applicable for observational studies of long duration, where there might be changes to study conduct over time.

In real‐world observational, retrospective studies, DMCs may have an evolving role in performing scientific validation of real‐world evidence and ensuring study integrity and credibility. Our DMC model for joint industry‐sponsored observational, retrospective safety studies has proven efficacy over 5 years and could be a precedent for others tackling similar pharmacovigilance activities.

## CONFLICT OF INTEREST

The sponsors have drafted and reviewed this manuscript and the DMC charter, as well as given financial support for establishing and maintaining the RDMC. None of the authors received honoraria for authoring the paper. Atheline Major‐Pedersen is an employee of and holds stocks in Novo Nordisk. Mary Kate McCullen is an employee and shareholder of AstraZeneca. Mary Elizabeth Sabol is an employee and shareholder of GlaxoSmithKline. Omolara Adetunji is a full‐time employee of Eli Lilly and owns shares in the company. Joseph Massaro received travel reimbursement and fees from AstraZeneca, Eli Lilly and Company, GlaxoSmithKline and Novo Nordisk A/S for serving on the Data Monitoring Committee. Alfred I. Neugut received travel reimbursement and fees from AstraZeneca, Eli Lilly and Company, GlaxoSmithKline and Novo Nordisk A/S for serving on the Data Monitoring Committee. Julie Ann Sosa received travel reimbursement and fees from AstraZeneca, Eli Lilly and Company, GlaxoSmithKline and Novo Nordisk A/S for serving on the Data Monitoring Committee. Anthony N. Hollenberg has received travel reimbursement and fees from AstraZeneca, Eli Lilly and Company, GlaxoSmithKline and Novo Nordisk A/S for serving on the Data Monitoring Committee.

## AUTHOR CONTRIBUTIONS

All authors approved the final version of the manuscript and take full responsibility for the content.

## Supporting information


**Data S1.** Supporting information.

**Data S2.** Supporting information.
