# Supplementary material for: A joint industry‐sponsored data monitoring committee model for observational, retrospective drug safety studies in the real‐world setting
Source: Pharmacoepidemiol Drug Saf. 2020 Nov 24;30(1):9–16. doi: 10.1002/pds.5172 (PMC8247341; doi:10.1002/pds.5172)
Supplement: Supplementary file 1 — Data S1. Supporting information. [file PDS-30-9-s001.docx]

# Precedence

- There is no guidance for data monitoring committees (DMCs) serving observational, retrospective studies.
- No experience/better practice regarding such DMCs has been shared.

# Indication for a DMC

- Enhancing study integrity, scientific validity and credibility of observational, retrospective safety studies is deemed the main indication for establishing a DMC, which is independent of study oversight and governance, to monitor accumulating safety data.
  - Especially relevant for long-term safety studies.

# DMC member selection process adapted to multiple sponsors

- Having a contract research organization (CRO) acting as facilitator between sponsors and between sponsors and potential DMC candidates may reduce process ownership of any one sponsor and simplify inter-sponsor dialogue regarding necessary compromises.
- In hindsight, sponsors can delegate identification of potential DMC candidates to the CRO or relevant independent professional body (e.g. American Thyroid Association [ATA] for thyroid-related safety studies), thereby further enhancing DMC sponsor independence and simultaneously simplifying inter-sponsor communication and consensus.

# DMC member selection

- DMC member expertise areas are adapted to the real-world data scenario: inclusion of epidemiologists, statisticians with real-world data/big data expertise.
- If we were to set up the DMC model today, we would consider also including patient advocates and data scientists.

5. DMC roles and responsibilities

- The DMC focuses on interpretation of cumulative safety data in order to assess potential safety patterns/signals in an independent manner.
- DMC independence from study conduct and oversight and from sponsors (if any) is essential for study integrity, scientific validity and credibility of observational, retrospective safety studies.

# Statistical analyses plan (SAP) finalization

- The SAP should be finalized only after all DMC members are on board and have reviewed and endorsed the SAP (as the SAP is the DMC´s working tool). The DMC continues, though, independent of study governance, study conduct and oversight, thus maintaining study integrity and credibility.

# Adjudication

- Establishing an adjudication process engaging the relevant DMC members can be considered if the definition algorithm to capture the event of interest does not have a widely accepted positive predictive value.
- For our study, the sponsor and Steering Committee, supported by the independent professional body (ATA), decided that the DMC would not perform adjudication of the outcome of interest (medullary thyroid cancer). All cancer cases captured from US state cancer registries are considered as true cases.

# Blinding

- Although blinding of treatment arm randomization is not applicable to observational retrospective studies, “blinding” can be used in multi-sponsored studies to maintain sponsor confidentiality.
- “Blinding” in this sense means other study committees/corporations remain blinded to the specific drugs. Each sponsor receives unblinded data related to its own drug from the CRO, enabling ongoing and timely pharmacovigilance activities.

# Meetings

- Having only DMC members meet at the data monitoring closed sessions (together with an external statistician) supports the DMC´s independence from sponsors and study government.
- Having sponsors call in to the data monitoring open session, instead of face-to-face meetings, reduces sponsor–DMC member contact, further enhancing DMC independence and study integrity and credibility.
- It is worth arranging a DMC kick-off meeting face to face with sponsors in order to “set the scene” by meeting each other prior to finalizing the DMC charter and the SAP.

1. Sponsor pre-DMC kick-off meeting (if multi-sponsored study)

- Can prove crucial for a successful DMC kick-off meeting.
- Its purpose is to ensure all sponsors are aligned to avoid inter-sponsor disagreements at the DMC kick-off meeting.

# DMC recommendations

- The recommendations known from randomized clinical trials are adjusted to support observational, retrospectively captured data:

# Communication flow

- The CRO proves pivotal in maintaining study integrity, scientific validity and credibility. All study-related communications between the DMC and sponsors and the DMC and Steering Committee are facilitated and documented by the CRO.

1. CRO operational oversight

- It is valuable to have a CRO responsible for data capture and data management-related processes when dealing with multiple study sponsors that have their own processes and Standard Operating Procedures.
