# Supplementary material for: A joint industry‐sponsored data monitoring committee model for observational, retrospective drug safety studies in the real‐world setting
Source: Pharmacoepidemiol Drug Saf. 2020 Nov 24;30(1):9–16. doi: 10.1002/pds.5172 (PMC8247341; doi:10.1002/pds.5172)
Supplement: Supplementary file 2 — Data S2. Supporting information. [file PDS-30-9-s002.docx]

Charter approval

On behalf of all the data monitoring committee (DMC) members:

Name:_______________________ (DMC chair)

Date: ________________________Signature: __________________________________
 (DD-MMM-YYYY)

The signatures below document the approval of the DMC charter:

| *Sponsor name* | *Approver name* | *Approver title or*  *approval committee* | *Signature* | *Date (DD-MMM-YYYY)* |
| --- | --- | --- | --- | --- |
|  |  |  |  |  |
|  |  |  |  |  |
|  |  |  |  |  |

Approval of the DMC charter is recorded by each sponsor´s approval committee in their meeting minutes, if applicable.

**Designated contact for the DMC**

| *Sponsor name* | *Designated contact´s name* | *Signature* | *Date (DD-MMM-YYYY)* |
| --- | --- | --- | --- |
|  |  |  |  |
|  |  |  |  |
|  |  |  |  |
|  |  |  |  |

Study information

**Introduction, background and objectives**

**Study design**

**Committees and corporations concerned with the conduct of the study**

**Consortium of study sponsors**

- Description
- Designated sponsor contacts and other key sponsor personnel are provided in [XX].

**Contract research organization (CRO):**

[XX] is the CRO acting on behalf of the consortium of study sponsors. Roles of the CRO in relation to the DMC include:

- Providing an external independent statistician.
- Ensuring documentation is available for DMC members on all requirements listed in the section “Data for DMC review”.
- Preparing and delivering data packages in a secure manner by approximately 3‑4 weeks before each DMC meeting.
- Preparing and archiving meeting minutes from all closed and ad hoc open DMC sessions.
- Supporting the DMC recommendations in written and signed form to the designated contact for each consortium sponsor, and subsequently to the Steering Committee chair **at end of business (EOB) on the 3rd working day after the closed session.**
- Maintaining contact information for the designated sponsor contacts and additional sponsor key personnel [Appendix XX].
- Maintaining contact information for the designated CRO personnel [Appendix XX].

**Study Administrative Committee**

The Administrative Committee will be responsible for the administrative management of the consortium. Members of this committee include the primary liaison(s) from each sponsor as well as the key stakeholders from the CRO. These functions and responsibilities are described in the study´s consortium agreement.

**Study Steering Committee**

The study´s Steering Committee provides scientific and academic leadership and oversees the conduct of the study. Roles and responsibilities of the Steering Committee are detailed in a separate charter. Specific Steering Committee accountabilities relating to the DMC are outlined below:

- Conduct regular reviews of the progress of the study.
- Steering Committee will have access, if requested, to blinded (as to the specific drug from the drug class) study data.
- Steering Committee chair will receive recommendation(s) from the DMC, via the CRO, **at EOB on the 3rd working day after the DMC´s closed session.**
- The Steering Committee chair may be invited to the ad hoc open session of the DMC meetings by the consortium sponsors.

The DMC

The DMC is established to perform an ongoing and independent evaluation of accumulated data from the study.

**DMC members**

The DMC constituted for this study is composed of X permanent voting members who cover relevant specialities.

Members of the DMC include [Appendix XX]:

- List of DMC member expertise areas.

The DMC statistician is responsible for providing statistical expertise and advice to the rest of the DMC members in the interpretation of the safety data and has voting rights. The DMC statistician shall not prepare the data package or additional analysis, as this is the task of the non-voting external statistician supplied by the CRO.

**Requirements for DMC members**

- At least a 5-year study commitment.
- Do not have conflicts of interest and are independent from the consortium sponsors, e.g. do not receive more than $XXXX annually compensation from consortium sponsors (besides their compensation for DMC-related activities), and are not shareholders in the respective companies.
- May not be a member of the study´s Steering Committee.
- Must complete an independence questionnaire annually in order to confirm there are no conflicts of interest with any of the participating sponsors.
- Received training for Good Clinical Practice, Good Documentation Practice and Collaborative Institution Training Initiative (CITI).

The DMC members may request assistance from additional ad hoc members if needed.

**DMC vacancies**

All DMC members are expected to serve until the study is complete. Should it be necessary for a member to resign, the member must submit an effective date of resignation in writing to the DMC chair, CRO and the sponsor-designated contacts. The study consortium, supported by the CRO, is responsible for filling DMC vacancies as soon as is feasible. Replacement of DMC members will be based on individual speciality competencies of the DMC.

In the case where a DMC member retires or discontinues participation in the DMC, the DMC member shall return or confirm destruction of all interim reports received, no later than the termination date according to the individual contract.

DMC tasks and responsibilities

The DMC should work in accordance with the relevant guidelines from Competent Regulatory Authorities.

The DMC responsibilities include making recommendations regarding the ongoing conduct of the study based on unbiased and independent review of unblinded safety data. The DMC shall review all the pertinent safety information and, using clinical and statistical judgement, make periodic recommendations to the designated contact for each consortium sponsor as described below. The DMC will function independently of all other individuals and entities associated with the conduct of the study.

**Specific roles of the DMC include:**

- Review content of the DMC charter.
- Define specific roles for each DMC member, concretize meeting dates and frequency together with the consortium sponsors.
- Review the statistical analysis plan (SAP).
- Determine what, if any, additional data or information each role requires.
- Meet face to face after implementation of the study; and thereafter one annual face-to-face meeting.
- Advise on protocol modifications proposed by the study consortium.
- Monitor compliance with current DMC recommendations.
- Assess the impact and relevance of external evidence (i.e. media debates, same-class drug information, literature).
- Receive and read annual FDA status report – for information.

**Making recommendations:**

The DMC will provide recommendations to the consortium sponsors at predefined intervals. The recommendations may not contain unblinded information that would unblind recipient(s) to specific treatment. The recommendations are:

- Continue unaltered.
- Continue with modifications.
- Report to FDA earlier than the next planned annual report submission.

The DMC members will make use of the available recommendation template [Appendix A] when making their recommendations to the sponsors.

The only point of direct contact for these interactions, as shown in Figure 1**,** will be the DMC designated contact for each of the consortium sponsors [Appendix XX] and the CRO, referred to as XXX from this point on [Appendix XX].

**DMC chair responsibilities:**

- Facilitating and summarizing DMC discussions.
- Ensuring documentation of all communication to the consortium sponsors on the conduct of the study or suggested changes to safety surveillance.
- Documenting meeting minutes from all closed DMC sessions (supported by the CRO).
- Providing DMC recommendations in written and signed form to all consortium sponsors, after each meeting as described in [Appendix XX] (supported by the CRO).
- Reaching agreement between the DMC members. If the DMC cannot achieve consensus, a vote shall be taken and the majority vote will prevail. If no majority consensus can be obtained, the chair has the final responsibility for making a recommendation.
- Determining the need for an open session, where blinded data are reviewed/discussed directly with sponsors (potentially also with the Steering Committee).
- Communicating to the sponsors, through the CRO, the need for an ad hoc open session with sponsors (and ad hoc invitation of Steering Committee chair).

**Stopping guidance/guidelines**

At present, no stopping guidance/guidelines are applicable for this study. However, the study is subject to implementation of stopping guidance if so imposed by the FDA.

DMC meeting structure

**Scheduled meetings**

A face-to-face kick-off meeting between the DMC members, CRO (including the CRO´s statistician) and the designated representatives and additional key personnel from each of the consortium sponsors was held for this study on _________ (date).

After the kick-off meeting, the DMC will have meetings at a minimum of every 12 months or more frequently, as needed. Additional key personnel may be invited.

It is preferred that the DMC has one face-to-face meeting (between DMC members, facilitated by the CRO) a year.

**Meeting structure**

The DMC meetings may be comprised of two sessions:

- An ad hoc open session, with attendance by DMC members, CRO (including an independent statistician) and designated contacts from each consortium sponsor where only blinded data will be reviewed and discussed. Ad hoc open sessions will take place when requested by the DMC chair or sponsors. Members from the Steering Committee may be invited for part of the ad hoc open session.
- A closed session, with attendance by DMC members (including the CRO external independent statistician) where unblinded (specific drug exposure and company are revealed) data are reviewed and discussed.

**Unscheduled meetings**

An unscheduled telephone conference or meeting can be suggested at the discretion of any member from the DMC or from designated contacts for each consortium sponsor.

**Meeting rules**

A minimum of five DMC members, including the DMC chair and the DMC statistician, is required to make a decision or recommendation considered valid by the consortium sponsors. Notwithstanding, in the case where a recommendation to modify the study is being considered and a member is absent, the DMC chair will seek the opinion of all committee members before a final recommendation is made.

If the DMC cannot achieve consensus, a vote shall be taken and the majority vote will prevail.

If no majority consensus can be obtained, the chair has the final responsibility for making a recommendation. The DMC chair will, after each data-review meeting, sign and date the DMC recommendation [Appendix A], on behalf of all the DMC members.

For votes taking place through teleconference, each DMC member must confirm their individual vote by submitting their vote in writing; this may be done electronically within the DMC folder on the secure portal to the DMC chair.

No details of the vote shall be included in the minutes from the ad hoc open session.

**Ad hoc consultation**

The DMC may request external ad hoc input in consultation with the consortium sponsors. The ad hoc member(s) must be identified in agreement with the consortium sponsors, and agreement should be documented by the designated contact from each consortium sponsor. The contracting process is handled by the CRO. The ad hoc member must be checked for conflict of interest as well as independence from consortium sponsors. Any changes in working procedures, including the request and identification of ad hoc consultation, shall be documented in the minutes from the ad hoc open session.

**Meeting documentation**

For each DMC meeting, at least two, potentially three, sets of documentation shall be prepared:

1. Ad hoc open session meeting minutes (when held)

- The CRO is responsible for preparing, finalizing, obtaining approvals and distributing meeting minutes to consortium sponsors approximately 4-6 weeks from the meeting. This includes obtaining review and active approval by all sponsors before minutes are forwarded to the DMC for review and obtaining timely DMC approval so the CRO can send the finalized minutes back to sponsors.
- The DMC chair will sign off the final version of the meeting minutes.
- The ad hoc open session meeting minutes may be used for communication internally and externally by each sponsor and to regulatory authorities, Ethics Committees and Institutional Review Boards on request, according to their internal policy.

1. Closed session meeting minutes

- The CRO will support the DMC chair to prepare, finalize, obtain approval and distribute meeting minutes to the DMC members approximately 4-6 weeks from the meeting.
- Minutes from the closed session shall contain a more detailed rationale and considerations for the DMC recommendation. As minutes from the closed session may contain unblinded information, these minutes are to be kept strictly confidential within the DMC/CRO and are not to be circulated otherwise. No data or fraction of data originating from any of the consortium sponsors can be used or made publicly available without the approval of the DMC and without pre-notice and approval of the members of the consortium.
- Minutes from the closed sessions will be formatted with a general section for all sponsors when the information is applicable to all drug class products. When the information is not applicable to all drugs from the drug class, the pertaining data and detailed rationale will be presented separately for each specific drug, e.g. separate sections or appendices per company. This procedure will facilitate reporting at the end of the study, as sponsors will be submitting their specific data in their final report at different time points, in order to fulfil their individual regulatory requirements. The minutes shall not reveal any information of a company-specific drug to other consortium sponsors. Company-specific drug data will only be revealed to the pertinent company, while all other data remain blinded as to type of specific drug exposure and company.

1. DMC recommendations (detailed below)

The DMC will provide recommendations to the consortium sponsors at predefined intervals. The recommendations may not contain unblinded information that would unblind recipient(s) to specific treatment. The recommendations may be:

- Continue unaltered.
- Continue with modifications.
- Report to FDA earlier than the next planned annual report submission.

The DMC members will make use of the available recommendation template [Appendix A] when making their recommendations to the sponsors.

The only point of direct contact for these interactions, as shown in Figure 1**,** will be the DMC designated contact for each of the consortium sponsors and the CRO.

DMC communication of recommendations/safety concerns

The DMC, through its DMC chair, will communicate recommendations to the designated contacts of the respective consortium sponsors via the CRO.

The DMC will have an indirect relationship to the Steering Committee chair through the CRO, in agreement with the sponsors.

**Figure 1: Communication flow for DMC recommendations/safety concerns to sponsors, CRO and Steering Committee**


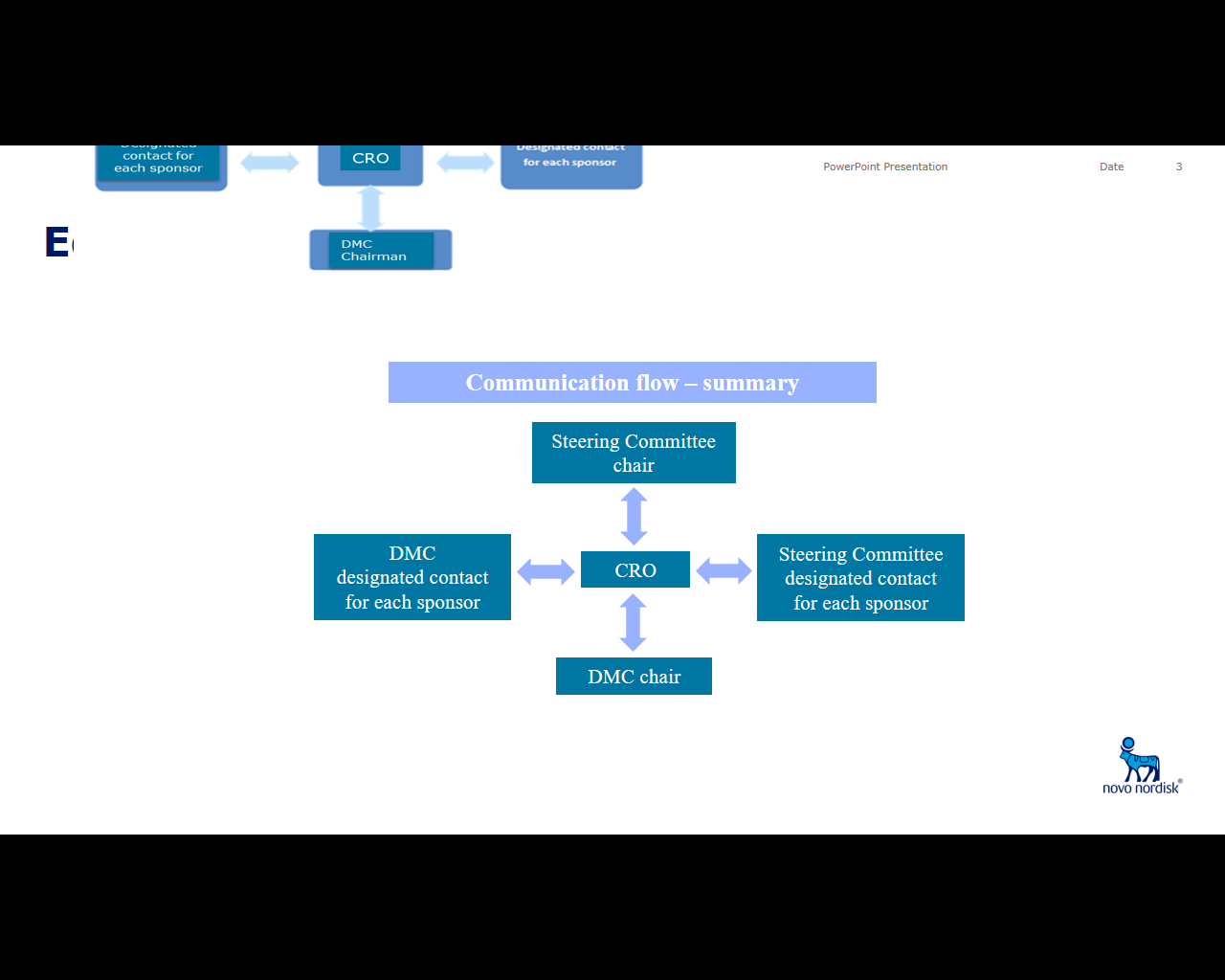


The recommendations of the DMC will be communicated to the designated contacts of the respective consortium sponsors via the CRO within 24 hours of the closed meeting. Recommendations of the DMC will be communicated to the Steering Committee chair, through the CRO, **at EOB on the 3rd working day after the closed session**.

If the DMC raises a safety concern, following its expert judgement, for the drug class or specific drugs from the drug class, the chair will inform all sponsors of this concern within 24 hours from the closed meeting, through the designated contact for each sponsor, facilitated by the CRO, in order to support surveillance activities of all consortium sponsors without unblinding sponsors to specific information for other than their own.

- **Recommendations** to continue the study unaltered, to continue the study with modifications, or to report to the FDA earlier than the next planned annual report submission will be made by the DMC chair to the designated consortium sponsor contact, via the CRO. The Steering Committee chair will be informed through the CRO, **at EOB on the 3rd working day after the closed session.** **Safety concerns** raised by the DMC´s own expert judgement, for one or more specific drug class, will be communicated by the DMC, always via the CRO, within 24 hours from the closed meeting, to the designated contact for each sponsor, in order to support surveillance activities of all consortium sponsors, without unblinding sponsors to specific information for the specific drug other than their own.

*It is up to the DMC´s discretion whether the initial communication to the sponsors, facilitated by the CRO, requires direct oral dialogue with the designated sponsor contacts, followed up with a written communication of the safety concern, or whether the written communication will suffice as the initial communication to each sponsor.*

*The written communication on the raised safety concerns will be communicated to the Steering Committee chair, by the CRO, at EOB on the 3rd working day after the closed session.*

- The designated contact for each consortium sponsor will facilitate the appropriate actions coordinated with the respective sponsors’ designated employees, according to each sponsor´s internal established procedures.
- As a minimum, when safety concerns/signals have been raised and/or recommendations to continue the study with modifications, or to report to the FDA earlier than the next planned annual report submission, a direct dialogue between the DMC chair, sponsor representatives (according to each company´s internal procedure), and the CRO shall take place before further actions and decisions are made.

Data for DMC review

**Sponsor-provided data/references**

Sponsors are responsible for providing DMC members with the following information via the CRO:

- Current version of the study protocol & SAP.
- US prescribing information.
- Investigator’s brochures (IB)/company core data sheet (if applicable for sponsor).
- Results of other pharmacovigilance activities conducted to characterize the potential risk of the study´s outcome of interest among patients exposed to the drug class.
- Any other significant information from any source a sponsor considers relevant to the safety outcome(s) of interest among users of the drug class.
- Upon revision and finalization of these documents, the designated consortium sponsor contact will provide the product-specific documents to the DMC, through the study´s CRO acting on behalf of the consortium sponsors.
- Annual study update reports submitted to the FDA – for information.

**Specific data packages**

**Blinded data**

Whenever study data are made available to the sponsors, all data pertaining to the event(s) of interest with reported drug-class exposure will be blinded regarding the consortium sponsors and specific drug exposure involved.

**Unblinded data**

- The relevant sponsor’s safety department will, however, receive unblinded data related to its own drug directly from the CRO.
- Available data will be collated and presented to the DMC members via the secure portal [see Appendix XX]. Unblinded data provided will include the specific drug´s generic and/or commercial names, and the sponsor´s name.
- Data packages presented in DMC-requested formats will be prepared and delivered via the secure portal by the external independent statistician (CRO) approximately 3-4 weeks prior to each DMC meeting.
- Quality control (QC) to ensure accuracy and completeness of the data package will be performed and documented by the CRO. Personal identifiable information will be de-identified in both blinded and unblinded data.

**End of study**

In order to fulfil regulatory requirements, when an individual company approaches their final report at the end of their participation in the study, the DMC chair (with the support of the CRO) shall provide the sponsor with the sponsor´s specific closed minutes. Closed minutes in addition to ad hoc open minutes will be provided approximately 4-6 weeks after the final meeting occurs.

**Ownership of data**

Each sponsor will govern access to their company-specific data.

**Maintaining records**

During the study, the DMC members shall store all other study-related documents according to industry standards after each meeting to ensure confidentiality – or delegate storage to the CRO.

Hard copies of signed minutes from the ad hoc open session and the DMC recommendations will be held by the CRO in the trial master file until the end of the study. These will be submitted to the designated contact for each consortium sponsor, and will be retained in accordance with each sponsor´s internal policies once the sponsor has ended their study participation.

Hard copies of the signed minutes from the closed session shall be retained by the DMC chair until the end of the study, unless otherwise agreed (e.g. delegate storage to the CRO).

Any notes or changes in DMC working procedures or notes on issues that may affect the working procedures – such as replacement of members or member conflict of interest – that do not result in a DMC meeting according to the rules in sub-section XX (“Making recommendations”) must be recorded in minutes and signed by the DMC chair.

After the study is reported, the DMC members shall return or confirm destruction of all interim reports received **no later than 3 months after the end of study** DMC meeting. The CRO must make a copy of all data packages prepared, including programming, and hand over this copy to the consortium sponsors at the end of the study for evaluation.

Independence/conflict of interests

The DMC members shall be independent from all consortium sponsors. They shall be free of financial interests that could be substantially affected by the outcome of this study. The DMC members will only have direct relationship with the designated contact for each consortium sponsors and with the CRO. The DMC will have no interaction, pertaining to the study, with those in the study leadership positions (including members of the Steering Committee) unless the contact has been arranged by the CRO in agreement with the consortium sponsors´ DMC designated contacts.

To ensure transparency, individuals invited to serve on the DMC must disclose any potential conflicts of interest, whether real or perceived, to the DMC chair, who in turn shall inform the consortium sponsors, according to the signed DMC member agreement. Conflict of interest can include professional interest or proprietary interest. No participant may have a vested interest in the outcome of the study.

On an annual basis, the DMC members will be requested to fill in and sign an independence questionnaire and disclose any changes in their status at the first DMC meeting. This will be documented in the meeting minutes from the ad hoc open session of that first DMC meeting.

# Appendix A

**Recommendation template for the DMC**

**Study title:**

**DMC meeting date:** DD-MMM-YYYY

**DMC members present: Date:** DD-MMM-YYYY

XX (DMC chair), XX, XX, XX, XX, XX,XX

**Purpose of meeting:** to review safety data with cut-off date DD-MMM-YYYY for the study (title)

**It was decided to recommend that the** (study title):

- Continue unaltered
- Continue with modifications
- Report to FDA earlier than the next planned annual report submission

Date of signature: ____________________________

(DD-MMM-YYYY)

Signature: _____________________________________________

Name of chair

Study´s (short) title - DMC chair

**Note to stakeholders:**

All contact to the DMC is handled by the study´s CRO (CRO´s name) and the designated contact for each consortium sponsor.
